# Recent advances in biosynthesis and regulation of strawberry anthocyanins

**DOI:** 10.1093/hr/uhaf135

**Published:** 2025-05-21

**Authors:** Xiaoyu Duan, Keru Wang, Renkun Tang, Jinying Liu, Kang Cheng, Guangtong Gao, Yuying Wang, Guozheng Qin

**Affiliations:** State Key Laboratory of Plant Diversity and Specialty Crops, Institute of Botany, Chinese Academy of Sciences, Beijing 100093, China; China National Botanical Garden, Beijing 100093, China; University of Chinese Academy of Sciences, Beijing 100049, China; State Key Laboratory of Plant Diversity and Specialty Crops, Institute of Botany, Chinese Academy of Sciences, Beijing 100093, China; China National Botanical Garden, Beijing 100093, China; University of Chinese Academy of Sciences, Beijing 100049, China; State Key Laboratory of Plant Diversity and Specialty Crops, Institute of Botany, Chinese Academy of Sciences, Beijing 100093, China; China National Botanical Garden, Beijing 100093, China; University of Chinese Academy of Sciences, Beijing 100049, China; State Key Laboratory of Plant Diversity and Specialty Crops, Institute of Botany, Chinese Academy of Sciences, Beijing 100093, China; China National Botanical Garden, Beijing 100093, China; University of Chinese Academy of Sciences, Beijing 100049, China; State Key Laboratory of Plant Diversity and Specialty Crops, Institute of Botany, Chinese Academy of Sciences, Beijing 100093, China; China National Botanical Garden, Beijing 100093, China; University of Chinese Academy of Sciences, Beijing 100049, China; State Key Laboratory of Plant Diversity and Specialty Crops, Institute of Botany, Chinese Academy of Sciences, Beijing 100093, China; China National Botanical Garden, Beijing 100093, China; University of Chinese Academy of Sciences, Beijing 100049, China; State Key Laboratory of Plant Diversity and Specialty Crops, Institute of Botany, Chinese Academy of Sciences, Beijing 100093, China; China National Botanical Garden, Beijing 100093, China; University of Chinese Academy of Sciences, Beijing 100049, China; State Key Laboratory of Plant Diversity and Specialty Crops, Institute of Botany, Chinese Academy of Sciences, Beijing 100093, China; China National Botanical Garden, Beijing 100093, China; University of Chinese Academy of Sciences, Beijing 100049, China

## Abstract

Cultivated strawberry (*Fragaria* × *ananassa*) is a kind of Rosaceae fruit crops grown worldwide. It is popularly consumed for its attractive color, juicy flesh, and nutrient content. The rich anthocyanin in strawberry fruits is responsible for its coloration. Anthocyanins are polyphenolic compounds, belonging to the four types of natural plant pigments. As important antioxidant secondary metabolites, anthocyanins substantially affect the internal quality and nutritional value of strawberry fruits. Here, we summarize the molecular mechanism underlying anthocyanin accumulation in strawberry fruits and discuss the ways to increase the content of anthocyanins in order to provide theoretical support for improving the color of strawberry fruits and enhance its commercial value by molecular biology methods.

## Introduction

Cultivated strawberry (*Fragaria* × *ananassa*), which belongs to the Rosaceae family, is widely planted in China, Europe, South America, and other tropical or subtropical areas. Because of its brilliant red color, sour–sweet flavor, and rich nutrition, strawberry owns the reputation of ‘Queen of fruits’. The quality evaluation of strawberry fruits includes inherent quality, such as texture, aroma, sugar and acid content, etc., and external quality, such as fruit size, shape, and color. Among them, fruit color is one of the core parameters to judge the quality of strawberry fruits. The characteristic color of strawberry fruits is mainly due to the accumulation of anthocyanins, which are flavonoid compounds that can be abundantly accumulated in stems, flowers, fruits, and seeds [1]. Anthocyanins are the largest and most important water-soluble natural pigments, also known as one of the four main categories of plant pigments, together with carotenoids, chlorophylls, and betalains [2]. To date, more than 600 anthocyanins have been detected in nature, contributing to the red, blue, purple coloration of flowers, fruits, and vegetables [3]. Anthocyanins cannot only resist biological and abiotic stress in plant, but also can remove reactive oxygen species (ROS) molecules produced under abiotic stress [4, 5]. Anthocyanins are also polyphenolic compounds with high antioxidant activity, which are easy to be absorbed and utilized by the human body, and generally considered to have anti-inflammatory activity, anti-cancer activity, and have potential in the prevention of cardiovascular diseases, obesity, and type II diabetes [6, 7]. Therefore, anthocyanin content is an important evaluation criterion to judge the quality of strawberry fruits, and it has become a trend to screen strawberries with high anthocyanin content.

Anthocyanins are composed of anthocyanidins and glycosides. There are six common anthocyanidins in higher plants: malvidin (Mv), pelargonidin (Pg), cyanidin (Cy), delphinidin (Dp), petunian (Pt), and peonidin (Pn). In addition to the different types of anthocyanidins, differences in the structure, number, and location of glycogroups, methyl groups, or acyl groups also lead to the diversity of anthocyanins, resulting in a wide variety of plant colors [8]. At present, cultivated strawberries have color phenotypes such as red, pink, dark red, white, and yellow, which are mainly determined by the different accumulation levels, species and distribution patterns of anthocyanins in fruits of different strawberry genotypes [9]. To date, 25 anthocyanin compounds and 4 proanthocyanidins have been identified in strawberry fruits [10]. There are six stages of development: small green fruits (S1), middle green fruits (S2), large green fruits (S3), white fruits (S4), initial ripening fruits (S5), and full ripe fruits (S6). Fruit color of the first three stages (S1–S3) appears to be green, then becomes white in the S4 stage, and changes to red from S5 stage to S6 stage. In the early stage of strawberry fruit development (S1–S4), proanthocyanidins are mainly accumulated in the fruits [11]. Together with fruit ripening, the proanthocyanidin content decreases, while the total anthocyanin content gradually increases, reaching the maximum value at S6 stage [12]. At S6 stage, pelargonidin-3-glucoside (Pg3G) and cyanidin-3-glucoside (Cy3G) are mainly accumulated. The content of Pg3G is the highest, accounting for 58.6%–93.6% of total anthocyanin content according to different strawberry varieties [13], and shows significant metabolic differences with the maturation of strawberry fruits.

Recently, the importance of anthocyanins in fruits has attracted increasing attention and the focus of anthocyanin research has gradually shifted from model plants to members of Rosaceae, such as apple, pear, cherry, and strawberry. This paper will discuss the advanced research on anthocyanins synthesis and regulation in strawberry, which may be helpful to provide reference for other Rosaceae members. We will focus on the molecular mechanism of anthocyanins accumulation in strawberry fruits from six aspects: anthocyanin biosynthesis, transcription factor regulation, transport, epigenetic regulation, hormone effects, and environmental factors.

## The anthocyanin biosynthetic pathway and the structural genes

**Figure 1:**
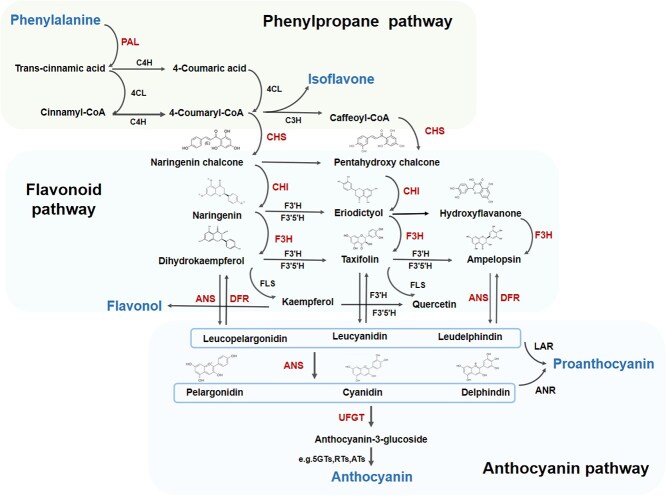
Schematic of anthocyanin biosynthetic pathway. The anthocyanin biosynthetic pathway belongs to a branch of the flavonoid biosynthetic pathway, which can be roughly divided into three pathways: phenylpropane pathway, flavonoid pathway, and anthocyanin pathway. The expression levels of some structural genes, such as *PAL*, *CHS*, *CHI*, *F3′H*, *DFR*, *ANS,* and *UFGT*, are closely related to the degree of fruit coloring at ripening stage. 5GTs, 5-glycosylation; RTs, rhamnosylation; ATs, acylation.

In 1995, Holton *et al.* summarized the biosynthetic pathway of plant anthocyanins in detail for the first time, based on studies on flower colors of pea, maize, and petunia [14]. At present, the anthocyanin biosynthetic pathway has been further clarified, with the most of the key structural genes in the pathway isolated. The anthocyanin biosynthesis belongs to a branch of the flavonoid biosynthetic pathway, which can be roughly divided into three pathways [15] (Fig. 1).

The first pathway is general phenylpropanoid metabolism. β-phenylacrylic acid is synthesized by phenylalanine, which is the synthetic substrate of anthocyanins, catalyzed by phenylalanine ammonia-lyase (PAL). β-phenylacrylic acid is then used to produce 4-coumarinyl-CoA under the catalysis of 4-coumarinyl-CoA ligase (4CL). The second pathway is flavonoid biosynthesis. Firstly, 4-coumaryl-CoA and three molecules of malonyl-CoA generate chalcone under the catalysis of chalcone synthase (CHS), constructing the basic skeleton of anthocyanins. Then, chalcone produces dihydroflavonols, co-catalyzed by chalcone isomerase (CHI) and flavanone 3-hydroxylase (F3H). Flavonoid 3′-hydroxylase (F3′H) and flavonoid 3′,5′-hydroxylase (F3′5′H) catalyze dihydroflavonols to form dihydroquercetins and dihydromyricetins, the precursors of anthocyanins. The third pathway is anthocyanin coloring. Colorless leucoanthocyanidins are formed under the catalysis of dihydroflavonol-4-dehydrogenase (DFR). Anthocyanin synthase (ANS) also called Leucoanthocyanidin dioxygenase (LODX) catalyzes the colorization of anthocyanidins. Finally, different types of glycoside groups, such as glucoside, galactoside, glycoside, arabinoside, xyloside, etc. are catalyzed by corresponding UDP-glucose-3-o-glucosyltransferase (UFGT) to attach to different positions of anthocyanidins to form a variety of stable anthocyanins. All the three stages above are completed in endoplasmic reticulum. Lastly, methylation on anthocyanins is completed by O-methyltransferase (OMTs), which produces malvacin, peonidin, and petunia. Under the catalysis of UFGT, the hydroxyl group of monosaccharide hemiacetal can combine with the hydroxyl group of another monosaccharide to form a diglycoside. The produced diglycosides can be further catalyzed by acyltransferases (ACTs) to generate acylated anthocyanins, in order to increase their stability and water solubility [16]. These end products are transported to the vacuole or other sites for storage, and exhibit a variety of colors under the influence of the vacuole PH, metal ions (such as Al^3+^ and Fe^3+^), and other coexisting pigments (such as flavonoids and flavonols) [17].

So far, most of the structural genes involved in the anthocyanin biosynthetic pathway in strawberry have been extensively studied in terms of classification and function. The expression levels of some structural genes, such as *PAL*, *CHS*, *CHI*, *F3*′*H*, *DFR*, *ANS*, and *UFGT*, are closely related to the degree of fruit coloring during the ripening stage and vary among different varieties. Genetically manipulation of these genes results in significant alterations in color phenotypes. Antisense suppression of *CHS* resulted in pink phenotype in cultivated strawberries (*Fragaria* × *ananassa* Duch.) with significant pigment loss [18]. In yellow woodland strawberry (*Fragaria vesca*), the expression of structural genes *C4H*, *CHS*, *CHI*, *F3*′*H*, *DFR,* and *ANS* was significantly down-regulated compared with that in red woodland strawberry, and transcriptional abundance of these genes was positively correlated with anthocyanin accumulation [19]. The expression levels of *FaCHI*, *FaDFR,* and *FaANS* are the highest in ‘HongYan’ strawberry (Benihoppe, red skin and red flesh), followed by ‘Xiaobai’ (red skin and white flesh), and lowest in ‘Snow White’ (white skin and white flesh) [12]. Silencing gene *FaANS, FaUFGT* [20], *FaCHS* [21], *FaF3*′*H* [22], *FaDFR* [23] by RNAi significant reduced anthocyanin levels in strawberry fruits, demonstrating that these genes play an important role in the anthocyanin biosynthetic pathway. Interestingly, an arginine–histidine mutation of F3H reduces its catalysis function and blocks anthocyanin biosynthesis, which leads to pink strawberry fruits [24]. Recently, a study demonstrated that loss-of-function mutation in an anthocyanidin reductase (ANR) activates anthocyanin biosynthesis in strawberry, resulting in the production of fruits with deep-red color [25]. Notably, it is FvDFR2 rather than FvDFR1 plays a key role for anthocyanin biosynthesis in strawberry petioles [26]. In developmental stage of strawberry fruits, the expression levels of *FaPAL1* and *FaC4H*, the key genes of phenylalanine metabolism pathway, increase. In the color transformation stage, the expression levels of *FaPAL2*, the upstream structural gene of phenylalanine metabolism pathway, and *FaCHS* and *FaCHI*, the upstream structural genes of flavonoid biosynthesis pathway, reach the peak. Mutation in *FvPAL2* results in a phenotype of light red fruits and yellow–green petioles in woodland strawberry ‘Ruegen’ [27]. After strawberry fruit ripening, the downstream structural genes in the anthocyanin biosynthetic pathway play a major role, while the upstream structural genes are down-regulated during fruit ripening [12].

## Effect of transport process on anthocyanin accumulation

The synthesis of anthocyanins takes place in the endoplasmic reticulum near the cytoplasmic side and then transported to the vacuole for storage through various distinct mechanisms. The stable acidic environment in the vacuole serves as a safeguard against the degradation of anthocyanins, allowing them to manifest diverse colors, and also preventing the high biochemical activity of anthocyanins from being toxic to cells [28]. In recent years, the decisive influence of transport process on anthocyanin accumulation in strawberry fruits has gradually attracted the attention of researchers. One of the most probable anthocyanin transport mechanisms in strawberry is that cytoplasmic GST catalyzes the covalent binding of glutathione (GSH) with anthocyanin, thereby generating GSH cross-linked complexes [29]. Subsequently, this complex is recognized by a GSH cross-linked binding pump situated on the vacuolar membrane. Through interactions involving the hydrophobic groups, this binding pump selectively attaches to anthocyanin, facilitating its transportation across the membrane and into the vacuole [30]. However, alternative studies have suggested that GST can directly bind to anthocyanin, transport it to the vacuole membrane, and then transport it across the membrane to the vacuole with the assistance of GSH cross-linked binding pump [30, 31]. GST protein has been shown to mediate anthocyanin transport in many species, such as *AN9* in petunia, *BZ2* in maize and *TT19* in arabidopsis, all of which encode a GST protein. After the functional deletion of the aforementioned gene, the content of anthocyanin in all tissues of the corresponding plants decreased significantly [29, 32, 33].

Recently, a reduced anthocyanin in petioles (*rap*) mutant was identified in chemically mutagenized population of Yellow Wonder, a white-fruited variety of the wild strawberry *F. vesca* [33]. *RAP* encodes a GST transporter that is essential for the coloration of foliages and fruits in strawberry [33]. Overexpression of *RAP* in Yellow Wonder induced a color alteration in the fruits from yellow to red [33], and this change was attributed to the heightened anthocyanin transport facilitated by the overexpression of *RAP*, which directly influenced fruit color independently of MYB10 and remained unaffected during ripening process. Transient knock-down of *RAP* also resulted in a significant decrease in fruit anthocyanin content in cultivated strawberry [33]. *RAP* acts downstream of MYB10 [33] and may function as a common downstream target of different regulatory pathways in anthocyanin metabolism. Enhanced anthocyanin transport can also shift early metabolic flux from proanthocyanidins to anthocyanin metabolic pathway in strawberry fruits [34]. In addition, *FvABCC8*, which encodes an ATP-binding cassette protein of type C transporter (ABCC transporter), is involved in strawberry vacuolar anthocyanin transport. The expression of *FvABCC8* is regulated by transcription factors MYB10 and bHLH33 [35, 36].

Although the anthocyanin transport pathway is still in the early stage of research with specific mechanisms yet to be fully elucidated, it provides a new perspective for further improvement of anthocyanin metabolic network and holds immense research potentiality.

## Effects of transcription factors on anthocyanin biosynthesis

The structural genes of strawberry anthocyanin biosynthetic pathway are regulated by a series of transcription factors, the most important of which is the ‘MBW (MYB-bHLH-WD40)’ ternary protein complex. It consists of R2R3-MYB transcription factors, bHLH transcription factors, and WD40 protein [37]. Till now, several MYBs related to fruit coloration in strawberry fruits have been identified, including MYB1, MYB5, MYB10, etc., of which FaMYB10 plays the dominant role in regulating anthocyanin synthesis [37–39]. bHLHs are also involved in anthocyanin biosynthesis, which can directly promote structural genes’ expression and anthocyanin accumulation by interacting with MYBs [37]. WD40 is a family of proteins with 4–10 random WD repeat domains. It has no catalytic function itself, but can interact reversibly with a variety of proteins to provide a platform for assembly of macromolecular protein–protein complexes and carry them to nucleus for further function [40]. Inhibiting the expression of *FaTTG1*, a WD40 protein gene, increased the content of anthocyanins in strawberry fruits [37].

### MYB

**Figure 2:**
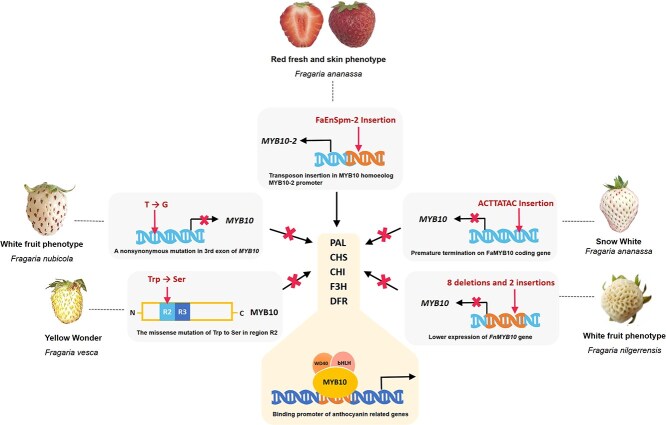
Mechanism of transcription factor MYB10 affecting phenotype of strawberries. MYB10 from different strawberry varieties plays an important role in anthocyanin accumulation. The missense mutation of Trp to Ser in region R2 of MYB10 in *Fragaria vesca*, a nonsynonymous mutation in the third exon of *MYB10* in *Fragaria nubicola*, and the mutations in *FnMYB10* promoter in *Fragaria nilgerrensis* are responsible for the low anthocyanin phenotype. The coding region of *MYB10* in ‘Snow White’ has ACTTATAC insertion, which leads to a decrease in the expression of *MYB10*, whereas a transposon FaEnSpm-2 insertion in the *MYB10* homoeolog *MYB10–2* promoter appears to be the key locus accountable for the natural variation in fresh and fruit skin. The expression of structural genes regulated by MYB10, such as *CHS*, *DFR*, etc., is therefore inhibited, resulting in the decrease in anthocyanin content and the difference in fruit phenotypes.

The plant transcription factor MYB is the largest transcription factor family that is widely present in plants. It regulates many physiological processes such as plant growth and development, secondary metabolism, and stress resistance. MYB proteins in plants are characterized by a highly conserved, DNA-binding R domain at N-terminal, which typically contains 1–4 amino acid repeats. According to the number of R domains, MYB transcription factors can be divided into four classes, of which R2R3-MYB members are the most numerous. They contain two R domains, and usually have a transcription activation domain on the C-terminal [39, 41]. Till now, more than 400 MYB genes and more than 380 R2R3-MYB genes have been found in strawberry [41], among which FaMYB10 is considered to be a key positive regulator of anthocyanin biosynthesis, and FaMYB1, FaMYB5 are key negative regulators. FaMYB10 and FvMYB10 from different strawberry varieties play an important role in anthocyanin accumulation. MYB10 can bind to the promoter region of *CHS2*, *DFR1,* and other key genes of the anthocyanin biosynthetic pathway, thus activating their expression [42]. Anthocyanin deficiency or overaccumulation phenotype can be obtained by different mutation of *MYB10* gene. In nature, various allelic mutations of *MYB10* are the main cause of natural differences in peel and pulp color of different strawberry varieties [43]. For example, overexpression of *FvMYB10* in woodland strawberries darkened fruit color, while inhibiting the expression of *FvMYB10* turned red fruits into white [44]. A gypsy-like reversal transposon was discovered in wild woodland strawberries with white fruits that truncates *FvMYB10* protein and disrupts anthocyanin biosynthesis [45]. The regulation of *FvMYB10* is tissue-specific, and the missense mutation of Trp to Ser in region R2 led to the Yellow Wonder phenotype in woodland strawberry fruits [19], but did not affect the accumulation of anthocyanin in petioles (Fig. 2). The content of anthocyanin in petioles is independently regulated by the homologous gene *FvMYB10L* [46]. In *Fragaria nubicola*, also known as Himalayan strawberry, a nonsynonymous mutation in the third exon of *MYB10* gene is responsible for its white fruit phenotype [47]. Mutations in *FnMYB10* promoter down-regulated its gene expression, which is responsible for the white fruit phenotype of *Fragaria nilgerrensis* [48]. Overexpression of *FaMYB10* in cultivated strawberries not only increased the level of anthocyanins in fruits, but also increased the level of anthocyanins in other tissues such as roots and leaves [49]. Studies have shown that the coding region of *FaMYB10* in ‘Xiaobai’ and ‘Snow White’ has GA insertion and ACTTATAC insertion, respectively. The premature insertion of the stop codon leads to the truncation of the entire FaMYB10 protein, causing a decrease in the level of anthocyanin content, which is responsible for the natural formation of their white flesh [50–52]. In addition, a CACTA-like transposon (FaEnSpm-2) insertion in the *MYB10* homoeolog *MYB10–2* promoter facilitates anthocyanin biosynthesis, which is the key locus responsible for the natural variation in the color of red fresh and fruit skin. Lack of this transposon leads to white flesh phenotype, while heterozygous lines displayed an intermediate phenotype [41]. Apart from stimulating anthocyanin biosynthesis, MYB10 can also facilitates the upregulation of the anthocyanin transporter glutathione S-transferase (GST), thereby fostering the transport of anthocyanins into vacuoles [33]. This regulatory action closely correlates with the ripening process of strawberry fruits [53].

Some members of strawberry MYB family negatively regulate anthocyanin biosynthesis. MYB1, which promotes anthocyanin biosynthesis in apples [54], is a typical transcriptional suppressor in strawberries. MYB1 shows high expression level only in mature or overripe strawberry fruits. It inhibits anthocyanin biosynthesis by competing bHLH sites in the MBW complex [55], and it also acts as a transcriptional suppressor of late anthocyanin biosynthesis genes such as *FLS*, *ANR,* and *F3*′*H* to balance anthocyanin level in late fruit ripening stage [56]. Certain investigations have indicated that in the ripening stages of strawberry ‘Maehyang’ with high anthocyanin content, the expression level of *MYB1* remained relatively stable. Conversely, in ‘Soelhyang’, which also has high anthocyanin content, there was an observed increase in *MYB1* expression level, whereas ‘Tokun’ with low anthocyanin content displayed no significant alteration in *MYB1* expression level [37]. Among woodland strawberries, the expression level of *FvMYB1* in the Yellow Wonder variety was significantly down-regulated compared with the red fruits [19]. Paradoxically, the expression level of *FcMYB1* in white Chilean strawberry (*Fragaria chiloensis*) was higher than that in red fruits. The decline in anthocyanin content observed in white Chilean strawberry fruits corresponded with a decrease in flavan-3-ol content subsequent to the down-regulation of *FcMYB1*. This suggests a plausible role for *MYB1* in facilitating the diversion of flavonoid precursors towards the biosynthetic pathway of proanthocyanidins at the branch point of the anthocyanidin/proanthocyanidin biosynthetic pathway [19]. *MYB1* expression is not regulated by MYB10 and vice versa. This indicates that MYB1 and MYB10 modulate anthocyanin biosynthesis mainly by affecting structural genes in the biosynthetic pathway [57].

In addition to MYB10 and MYB1, some other members of MYB family are also involved in the regulation of strawberry anthocyanin biosynthesis. FaMYB7 promoted anthocyanin accumulation by affecting *FaUFGT* expression [50]. FaMYB5 [37], MYB39, and MYB86 [58] may be potential positive regulators of anthocyanin biosynthesis. In addition, FaMYB28, FaMYB54, and FaMYB576 may be involved in regulating anthocyanin biosynthesis in strawberry [59]. Moreover, some transcription factors exhibit a shared regulatory influence on the accumulation of anthocyanins or proanthocyanidins. For example, FaMYB9/FaMYB11 can form terteric complexes with FabHLH3 and FaTTG1, and up-regulate *LAR* and *ANR* to increase the content of proanthocyanidins, competing with the anthocyanin biosynthetic pathway [39].

### bHLH

Basic helix–loop–helix (bHLH) is the second largest family of transcription factors in plants, consisting of a highly conserved bHLH domain of 60 amino acids [60]. Although bHLH has no independent function in regulating anthocyanin biosynthesis itself, it can interact with transcription factors such as MYB to regulate anthocyanin accumulation. For example, FvbHLH33 can interact with FvMYB10 to activate *FvDFR* and *FvUFGT* promoters, facilitating anthocyanin accumulation [49]. FabHLH3 and FabHLH3∆ promote proanthocyanidin biosynthesis but inhibit anthocyanin biosynthetic pathway by interacting with four MYBs [39]. FabHLH25 has a robust capacity to interact with MYB113, Jasmonate ZIM-domain 5 (JAZ5) and JAZ6 proteins to form MBW complex to regulate the expression of genes related to the proanthocyanidin biosynthetic pathway. FabHLH98 may co-activate anthocyanin biosynthetic pathway with MYB75/PAP1 [61]. Studies have shown that seven FabHLH transcription factors (FabHLH17, FabHLH25, FabHLH27, FabHLH29, FabHLH40, FabHLH80, and FabHLH98) are involved in anthocyanin biosynthesis and hormone signal transduction pathways in strawberry peel and pulp [61].

### Other transcription factors

In addition to MYB, bHLH, and WD40 proteins, MADS, mitogen-activated protein kinase (MAPK), B-box (BBX), and other transcription factor families also play important roles in regulating anthocyanin metabolic pathway. They may interact with MBW complex, influencing its upstream or downstream, thereby contributing to the comprehensive construction of anthocyanin regulatory network. The MADS-box transcription factor FaMADS1a inhibits anthocyanin accumulation in strawberry fruits by repressing the expression of *FaPAL6*, *FaCHS*, *FaDFR,* and *FaANS* [62]. FaBBX22 of the BBX transcription factor family is a facilitator of anthocyanin by promoting both biosynthesis and transportation of anthocyanins, particularly under light-exposed conditions [63]. EOSINTE BRANCHED 1, CYCLOIDEA, and PROLIFERATING4 CELL FACTORS 9 (FvTCP9) regulate anthocyanin biosynthesis through interaction with MYC1 [64]. FaGAMYB (gibberellin-induced MYB) can elevate the concentration of ABA and sucrose consequently bolstering the expression of *FaMYB10* in strawberry, thus promoting anthocyanin biosynthesis during ripening [65]. APETALA2 (FaAP2) and ripening inducing factor (FaRIF) can also enhance anthocyanin biosynthesis during ripening [66, 67]. Silencing *FaMADS9* gene inhibited normal development and ripening of strawberry achenes, thereby inhibiting anthocyanin biosynthesis [68]. Some families of transcription factors, such as WRKY and bZIP families, have been recognized for their regulatory roles on anthocyanin metabolism in various plant species. However, their specific impact on anthocyanin biosynthesis in strawberry has yet to be distinctly elucidated, warranting further investigation and clarification [69, 70]. Through transcriptome-related network analysis, additional transcription factors potentially involved in anthocyanin biosynthesis have been identified [71], indicating that the comprehension of the gene regulatory network of strawberry anthocyanin metabolic pathway remains incomplete and needs to be further deepened.

## Epigenetic effects on anthocyanin accumulation

**Figure 3:**
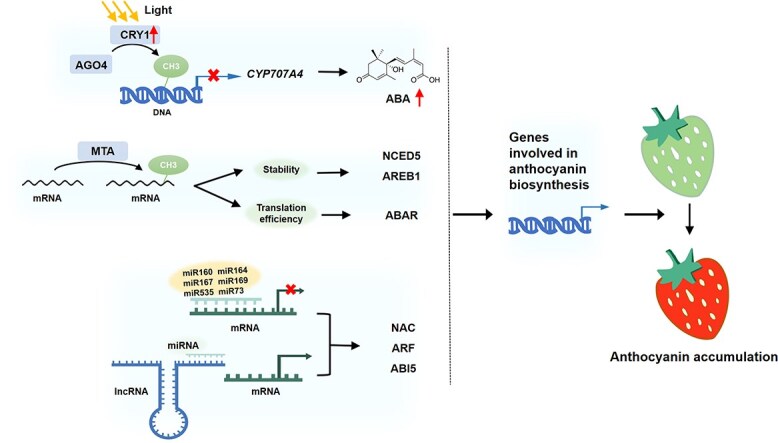
Mechanism of epigenetic regulation in controlling anthocyanin accumulation. Light induces CRY1, which can affect AGO4 binding to the promoter of *CYP707A4*, a key ABA catabolism gene, and increase its methylation levels. This promotes ABA accumulation and the increase in anthocyanin content. RNA m^6^A methylation enhances mRNA stability of *NCED5* and *AREB1*, the pivotal genes involved in ABA biosynthesis and signaling pathway, and improves the translation efficiency of ABAR, an ABA receptor, thus promoting the ripening of strawberry fruits and the accumulation of anthocyanins. Various non-coding RNAs, such as miRNAs and lncRNAs, play an important role in anthocyanin metabolism. miR160, miR164, miR167, miR169, miR535, and miR73, along with their respective target genes, modulate key transcription factors. lncRNAs can competitively bind to miRNAs, thereby blocking the inhibitory effect of miRNAs on their target mRNAs, thus participating in anthocyanin regulation.

Epigenetics, which refers to heritable changes in gene expression without alterations in nucleotide sequence, has emerged as a prominent focal point in the field of biological science in recent years. Anthocyanin biosynthesis in strawberry can be affected by epigenetic inheritance including DNA methylation modification, histone modification, and non-coding RNA modification (Fig. 3). Among them, DNA methylation regulates gene transcription through promoter modification and genome stability, histone modification inhibits or promotes gene expression according to different modification sites and types, while non-coding RNA functions by targeting specific mRNA molecules [72].

In the anthocyanin metabolic pathway, the promoter region methylation of transcription factors inhibits their expression, thus down-regulating anthocyanin content. For example, light induces cryptochrome 1 (CRY1), which can affect argonaute 4 (AGO4) binding to the promoter of *ABA 8′-*hydroxylase (*CYP707A4)*, and increase its methylation levels. This promotes ABA accumulation and the increase in anthocyanin content [73]. DNA demethylation in strawberries up-regulated the transcription levels of *PAL, 4CL, CHS, CHI*, and *F3′H* genes related to anthocyanin biosynthesis [73, 74]. Injecting DNA methylation inhibitor 5-azacytidine (AZ) into the whole strawberry fruits inhibited anthocyanin accumulation [74, 75]. Both DNA methylation and histone methylation contribute to ABA-mediated signaling pathways, thereby influencing the onset of fruit ripening. Through inhibiting ABA biosynthesis and augmenting ABA degradation, AZ-induced hypomethylation notably diminishes ABA accumulation. Consequently, this inhibition hampers ABA-mediated signaling pathways associated with ripening, thereby curtailing anthocyanin production and transport, and significantly delays the discoloration and aging of postharvest strawberries [75, 76]. Furthermore, RNA N^6^-methyladenosine (m^6^A) has been observed to enhance the mRNA stability of *9-*cis-epoxy-carotenoid dioxygenase *5* (*NCED5)* and *ABA-*responsive element binding proteins *1* (*AREB1*), the pivotal genes involved in the ABA biosynthesis and signaling pathway. The m^6^A modification also improves the translation efficiency of ABAR, an ABA receptor, thus promoting the ripening of strawberry fruits and the accumulation of anthocyanins [77]. More recently, the FvABF3-FvALKBH10B-FvSEP3 cascade was reported to regulate anthocyanin accumulation in strawberry fruit. ABA-responsive element binding factor 3 (FvABF3), a key regulator of ABA signaling, activates the expression of *FvALKBH10B* encoding an RNA m^6^A demethylase, which positively modulates the mRNA stability of *SEPALLATA3* (*FvSEP3*) through m^6^A demethylation. In turn, FvSEP3 regulates the expression of genes associated with biosynthesis of ABA and anthocyanin, such as *FvNCED1* and *FvCHS* [78].

Various non-coding RNAs, such as miRNAs and lncRNAs, also play an important role in anthocyanin metabolism. *FvMYB1* was identified to have two miRNA-regulated target sites [79]. Decreasing the expression levels of these two miRNAs markedly enhanced the expression of *MYB1* in fruits. Consequently, this upregulation of *MYB1* expression led to the inhibition of anthocyanin biosynthesis [79]. A large number of studies have demonstrated that miRNAs participate in ABA-mediated signaling pathways and thus play a crucial role in strawberry fruit ripening. For example, miR160, miR164, miR167, miR169, miR535, and miR73, along with their respective target genes, modulate key transcription factors such as NAC (NAM, ATAF1/2, CUC2), ARF, and ABI5 [80, 81]. miR5290 and Fa_novel23 have been found to enhance the biosynthesis of anthocyanin in strawberry fruits by suppressing the expression of anthocyanin negative regulatory factors FaMADS1 and FaKFB under the influence of ABA [58, 82]. Meanwhile, a total of 50 601 potential lncRNAs were screened and identified in octoploid strawberries [51]. It has been demonstrated that lncRNAs can competitively bind to miRNAs, thereby blocking the inhibitory effect of miRNAs on their target mRNAs, thus participating in anthocyanin regulation. Consequently, a comprehensive regulatory network, involving interactions between lncRNAs, miRNAs, and mRNAs, has been established to govern anthocyanin regulation in strawberries [51]. All in all, epigenetics holds significant research potential in elucidating the regulatory mechanisms of anthocyanin metabolism.

## Effects of hormone regulatory network on anthocyanin accumulation

Plant hormones play a crucial role in the regulation of strawberry anthocyanin metabolism, and their influence on anthocyanin accumulation is mainly realized through fruit ripening process. As a typical non-climacteric fruits, strawberries have been extensively investigated regarding the signaling pathways involved in ripening. Given that the alteration in strawberry fruit color is a readily observable phenomenon during ripening, anthocyanin accumulation is intimately associated with the ripening process [83]. Hormones such as abscisic acid (ABA), auxin (IAA), gibberellin (GA), jasmonic acid (JA), and ethylene actively participate in both the ripening and coloring processes of strawberry fruits [53]. In addition to the direct effects of these hormones themselves on maturation regulation, the regulatory network orchestrated by ABA, involving various ripening hormones, sucrose, and other signaling molecules, often exerts a more significant influence [84].

ABA serves as the primary regulatory hormone in the ripening process of non-climacteric fruits. It plays integral roles in softening, aroma biosynthesis, and fruit coloring, exerting influence over various aspects of fruit maturation and development [57, 85]. ABA can increase anthocyanin content in strawberry fruits through hormone signaling and biosynthesis of secondary metabolites [84]. The biosynthesis and signaling of ABA are primarily regulated by the key gene *FaNCED1*. After silencing *FaNCED1*, endogenous ABA content decreased, resulting in white phenotype of strawberry fruits [86]. ABA inhibits the expression of negative regulators of anthocyanin biosynthesis and promotes anthocyanin accumulation by inducing miRNAs biosynthesis [58]. ABA can also regulate the expression levels of *FaMYB10* and *FaMYB1*, while simultaneously enhance carrier-mediated transvesicular and plasma membrane transport mechanisms. This multifaceted regulatory action ultimately influences anthocyanin biosynthesis within strawberry fruits [52, 87].

During the early stages of development, IAA and GA synthesized in strawberry achenes regulate the development of flower receptacles [88]. Therefore, effective communication between the achene and receptacle becomes crucial. In later stages, the maturation of the receptacle relies on a reduction in auxin content and the biosynthesis of local ABA [86]. When IAA concentration is low, the auxin response factors (ARF) family proteins harbor no transcriptional regulation function due to the formation of heterodimer with the Aux/IAA family proteins. When IAA content increases, the higher IAA induces the ubiquitination and degradation of Aux/IAA proteins, which releases ARF proteins. The released ARFs such as FaARF2 can bind to *FaCHS* promoter and inhibit its expression, thus inhibiting anthocyanin accumulation [89, 90]. Besides, ABA can synergistically regulate fruit ripening with IAA. In early stage, ABA-mediated IAA transportation from achenes promotes receptacle growth and expansion, while in late stage, ABA inhibits IAA biosynthesis to facilitate receptacle ripening [91]. The ripening induction factor FaRIF plays a pivotal role in promoting the development and ripening of strawberry fruits, as well as facilitating the accumulation of anthocyanin. FaRIF orchestrates the interaction of plant hormones, particularly IAA and ABA. Furthermore, FaRIF holds significance in inducing anthocyanin biosynthesis in achenes and possesses the capability to independently regulate anthocyanin biosynthesis in the flower stock, regardless of achene development [67]. Another study demonstrated that FaGAMYB, situated upstream of the ABA signaling pathway, can modulate the interaction between GA and ABA throughout the ripening process of strawberry flower receptacles, thus affecting anthocyanin biosynthesis [65]. JA is positioned downstream of the ABA signal and plays a positive role in regulating the expression of genes associated with anthocyanin metabolism, including *CHS, CHI, F3H, PAL, and ANS* [92]. Bioactive jasmonoyl-L-isoleucine (JA-Ile) can induce the interaction between F-box CORONATINE INSENSITIVE 1 (COI1) and JASMONATE-ZIM DOMAIN PROTEIN (JAZ) to form a co-receptor complex, leading to ubiquitination-mediated degradation of JAZ proteins [93], which act as a repressor of ripening-related transcription factors, including JA-regulator FaMYC2 and FabHLH3. JA-induced degradation of JAZ proteins releases FabHLH3 to form FaMYB10-FabHLH3 complex, thus promoting anthocyanin biosynthesis [20, 94]. Application of exogenous methyl jasmonate (MeJA) substantially enhances anthocyanin accumulation [92]. However, MeJA results in a reduction in ABA content, indicating a potential antagonistic relationship between MeJA and ABA in the regulation of strawberry fruit ripening [92].

Strawberries have traditionally been categorized as typical non-climacteric fruits due to their minimal ethylene production during ripening and limited response to exogenous ethylene [83]. Nevertheless, in recent years, numerous studies have unveiled the involvement of ethylene in the later stages of strawberry ripening [95]. Ethylene has been shown to augment the activity of PAL, an enzyme essential for anthocyanin metabolism [96], while also influencing cell membrane permeability and expediting the cycling and accumulation of sucrose, and thereby promote anthocyanin biosynthesis [97]. Additionally, ABA has the ability to enhance the sensitivity of strawberry fruits to ethylene, enabling its functionality even at low endogenous levels [98]. It should be noted that, although served as a marker for the ripening of strawberry fruits with red skin or red flesh, the accumulation of anthocyanins is not essential for the ripening of white-fruited varieties. During strawberry fruit ripening, the changes in sugar driven by sucrose transporters such as FaSUT1 [99], aroma driven by nerolidol synthase and eugenol synthase [100, 101], and texture driven by pectate lyase [102] are independent from those of anthocyanins. Therefore, fruit can ripe without the accumulation of anthocyanins in the white-fruited varieties.

## Effects of exogenously applied compounds on anthocyanin accumulation

Exogenous application of certain substances can influence the anthocyanin metabolism of strawberry fruits. During the later stages of strawberry fruit development, sucrose plays a crucial role as it serves as a vital substrate for the biosynthesis of numerous pigments and aromatic compounds. Sucrose is the main source of anthocyanin biosynthesis, and lack of sucrose will delay the accumulation of fruit pigment [103]. Beyond its function as a carbon source, sucrose also serves as a signaling molecule, instigating fruit maturation and ABA accumulation. This pivotal role of sucrose extends to regulating transcription factors and metabolic enzymes involved in anthocyanin biosynthesis at both transcriptional and post-transcriptional levels, thus exerting a profound influence on the ripening and coloring processes of the fruits. The exogenous application of sucrose has been found to induce ABA accumulation in strawberry fruits, thereby promoting anthocyanin biosynthesis [104]. *FaSUT1* gene encodes a major carrier protein responsible for sucrose transport during fruit development, and overexpression of *FaSUT1* gene can increase sucrose and ABA level and accelerate anthocyanin biosynthesis [86, 99]. Sucrose and ABA also play a regulatory role in modulating the expression of the transcription factor ABA-stress-ripening (ASR). ASR serves as a significant factor in hastening the maturation and anthocyanin accumulation of strawberry fruits under stress conditions, and alterations in its gene expression impact the expression of anthocyanin biosynthesis-related genes such as *CHS, CHI, F3H, DFR, ANS,* and *UFGT*. These findings highlight the importance of cross-signaling between ABA and sucrose in governing anthocyanin biosynthesis [85].

Proanthocyanidins have been observed to inhibit the expression of genes related to anthocyanin biosynthesis, resulting in decreased anthocyanin accumulation and a slower color change in strawberries. Additionally, proanthocyanidin treatment has been found to increase the content of endogenous proanthocyanidins, ABA, and sucrose to a certain extent. Due to the competitive nature of proanthocyanidin and anthocyanin biosynthesis within the phenylalanine pathway, the overexpression of regulators of proanthocyanidin biosynthesis in strawberry can lead to a reduction in anthocyanin content [105]. Conversely, the silencing of anthocyanin glycosyltransferase FaGT1 redirects anthocyanin towards the proanthocyanidin pathway in the flavonoid pathway. Moreover, during the early stages of strawberry fruit development, silencing of anthocyanidin reductase (ANR), a key enzyme in proanthocyanidin biosynthesis, results in decreased proanthocyanidin content and increased anthocyanin content [106]. The injection of exogenous melatonin during the light green stage of strawberry (*Fragaria annanaca* cv.) has been shown to enhance the accumulation of endogenous melatonin. This elevation in melatonin levels correlates with increased expression of the *GAMYB* gene and reduced expression of the *SnRK2.6* gene, subsequently leading to heightened expression of *PAL* and *CHS* genes, facilitating the accumulation of anthocyanins in strawberries [107]. Treatment with exogenous terpene-4-ol has been found to upregulate the expression of genes associated with the pentose phosphate, phenylalanine, and flavonoid pathways, as well as the transcription factor MYB10. This regulatory effect ultimately promotes anthocyanin accumulation by engaging in the sucrose signaling pathway [108]. In summary, emerging evidence suggests that the ripening and coloration of strawberry fruits are orchestrated by a synergistic interplay of multiple signaling pathways.

## Influence of external environmental factors on anthocyanin accumulation

In addition to endogenous factors, anthocyanin accumulation is also affected by external factors such as light, temperature, humidity, etc., especially in the aspect of environmental stress. Numerous studies have underscored the pivotal role of anthocyanins as natural stress alleviators, both in response to abiotic and biological stresses. The biosynthesis of anthocyanins under stress conditions is tightly regulated by the MBW complex [109]. Abiotic stresses known to induce anthocyanin accumulation include ultraviolet radiation, drought, cold, and elevated levels of CO_2_, among others.

### Light

Light plays a critical role in the accumulation of anthocyanin in strawberry fruits. Compared to open-air cultivation, the anthocyanin content in strawberries grown under shading film is notably reduced. However, the anthocyanin content remains unaffected in strawberries cultivated with UVB transparent film [110]. Sufficient light plays a pivotal role in regulating MYB10 and stabilizing the FvMYB10 protein at both translational and post-translational levels. Moreover, light exposure fosters the expression of genes involved in anthocyanin biosynthesis, including *CHS*, *F3′H*, *CHI*, and *DFR* [111]. As various photoreceptors can discern distinct spectral ranges, different photoeffects such as photoperiod, light intensity, and light quality exert varying effects on anthocyanin biosynthesis [112]. Anthocyanin biosynthetic genes are tightly regulated by both light intensity and quality. High light intensity has been observed to induce the expression of *MYB10*, while *MYB1* expression remains unaffected [49]. Various wavelengths of light can elicit distinct expression patterns of R2R3-MYB transcription factors, consequently leading to significant variations in anthocyanin content [112]. When strawberry fruits at the white stage were subjected to light treatments of varying wavelengths, including blue, green, red, and white light, anthocyanin content, particularly under blue light treatment, significantly increased compared to dark conditions and was notably higher than that of green and red light treatments [113, 114]. Additionally, after 4 days of treatment, the expression of *FaCHS* and *FaF3′H* genes exhibited significant upregulation [113, 114]. Red light treatment has been shown to selectively enhance the accumulation of pelargonidin-like anthocyanins via the ABA signaling pathway, and accelerate endogenous ABA biosynthesis and deglycosylation processes [106]. Cultivation under various color and light quality selective films revealed distinct effects on anthocyanin content in strawberry fruits. Specifically, strawberry fruits treated with red and yellow films exhibited significantly higher anthocyanin content compared to those under white film. Conversely, strawberry fruits treated with green and blue films showed significantly lower anthocyanin content compared to white film. This suggests that shorter wavelengths, such as green and blue light, exert the most pronounced influence on anthocyanin accumulation [115].

Moreover, light quality exerts regulatory effect over anthocyanin accumulation by influencing the expression of photoreceptors such as Phytase (PPHY), CRY, and PHOT, as well as light signal transduction elements including constitutively photomorphogenic 1 (COP1), elongated hypocotyl 5 (HY5), and suppressor of PHYA-105 (SPA). It was shown that the red/far-red light photoreceptor FvePhyB can regulate anthocyanin accumulation in woodland strawberry by modulating the expression levels of *FveCHS1*, *FveF3H*, *FveDFR2*, *FveANS*, *FveUFGT*, and *FveMYB10* [116]. The blue light signal transduction module FaCRY1-FaCOP1-FaHY5 holds the ability to regulate anthocyanin accumulation in cultivated strawberry [117]. Additionally, light quality modulates the expression of transcription factor genes such as *MYB*, *bHLH*, and *WD40*, along with structural genes like *CHS*, *ANS*, and *DFR* within the anthocyanin biosynthetic pathway [115]. The protein complex, FaBBX22-FAHY5, akin to the MBW complex, operates to enhance the biosynthesis of anthocyanins in strawberries under light conditions [63].

Anthocyanin functions as a robust antioxidant, and its accumulation serves as a shield against the detrimental effects of ultraviolet (UV) light on strawberries. Mild UV irradiation prompts the upregulation of genes including *CHS*, *CHI*, *DFR*, *FLS*, and *UFGT*, mediated by elevated levels of ABA and ASR leading to the accumulation of anthocyanins, bolstering the fruit’s defenses against UV radiation [118].

### Temperature

Temperature represents another crucial environmental determinant influencing anthocyanin biosynthesis in strawberries. Lower nighttime temperature impedes respiration and facilitates sugar accumulation in strawberry fruits. Consequently, pronounced temperature difference between day and night yields superior coloring effects in strawberries [119].

Nevertheless, excessively low temperatures can yield unfavorable fruit coloration, substantially diminishing its commercial appeal [120]. The expression of genes involved in anthocyanin biosynthesis demonstrates sensitivity to temperature fluctuations. In particular, the transcriptional regulation of the SnRK2.6 signaling molecule, downstream of the ABA pathway, acts to downregulate the expression of anthocyanin biosynthetic genes under low-temperature conditions [121]. Low temperature can also inhibit anthocyanin accumulation in strawberry fruits through mitogen-activating protein kinase3 (FvMAPK3)-mediated phosphorylation of FvMYB10 and FvCHS1, leading to the decrease in transcriptional activity of FvMYB10 and proteasome-mediated degradation of FvCHS1, respectively [120]. Under the stress of low temperatures, anthocyanin accumulates within epidermal cells, thereby diminishing the osmotic potential of cells and providing protection against freezing-induced damage to plants. In contrast to the high cold-tolerant octoploid strawberry cultivar ‘Jonsok’, another octoploid strawberry cultivar ‘Frida’, which exhibits limited cold tolerance, displays reduced expression of cold-tolerance-related proteins. However, it effectively mounts a response to cold stress through elevating the expression of genes involved in the anthocyanin biosynthetic pathway, such as *CHS*, *F3′H*, and *DFR* [122]. Transgenic strawberry with heightened expression of the dehydroresponsive element binding protein (DREB) transcription factor RdreB1BI exhibited upregulation of key enzyme genes involved in anthocyanin biosynthesis during early developmental stages, thereby fostering anthocyanin accumulation, and thus improving the cold hardiness of the *RdreB1BI* transgenic strawberry [123].

### Other environmental factors

Besides light and temperature, other environmental factors exert influence on anthocyanin metabolism. Under conditions of water stress, there is an observed elevation in the expression of *FaMYB10* alongside an increase in anthocyanin content within strawberry fruits [112]. Phosphate deficiency in soil induces osmotic stress, halting cell division, and prompting the accumulation of phenylalanine, which subsequently diverts towards the anthocyanin metabolic pathway. This stress condition reduces cell density, fostering light penetration and enhancing anthocyanin content. Additionally, a high concentration of sucrose can act as an osmotic agent, accumulating phenylalanine and augmenting anthocyanin production under osmotic stress [124]. Both water stress and osmotic stress can induce ABA biosynthesis, triggering ABA signaling pathways that promote anthocyanin accumulation. Upon detachment, strawberry fruits experience enhanced respiration rates, elevated ABA and ethylene levels, accelerated IAA degradation, and generally decreased expression levels of *FaMYB1*, *FabHLH3*, and *FaTTG1*. These changes lead to the accumulation of more anthocyanins, resulting in rapid color transformation from white to full red within 1–2 days [125]. Under 20% CO_2_ stress, the expression of *PAL*, *C4H*, *CHS,* and other anthocyanin biosynthetic pathway genes in strawberry fruits decreased, leading to a significant decrease in anthocyanin concentration [126]. Indeed, understanding how environmental factors influence anthocyanin production in strawberries is crucial for developing strategies to enhance coloration in adverse conditions.

By elucidating the mechanisms underlying anthocyanin regulation in response to various environmental stresses, researchers can potentially breed strawberry varieties that maintain vibrant coloration even under challenging growing conditions. This knowledge can ultimately contribute to improving the commercial value and resilience of strawberry crops.

## Conclusions and perspectives

This review highlights the intricate process of anthocyanin biosynthesis in strawberries, which initiates from phenylalanine and involves a cascade of enzymatic reactions catalyzed by various enzymes encoded by structural genes. The glycosylation of anthocyanins by UFGTs leads to the formation of diverse anthocyanin compounds, which are then transported to the vacuole for storage and accumulation. This comprehensive understanding of anthocyanin metabolism sheds light on the factors influencing fruit coloration and provides insights into strategies for enhancing anthocyanin accumulation in strawberries. The acknowledgment of anthocyanin’s regulation by epigenetics, endogenous hormones, and environmental factors underscores the complexity of its biosynthesis in strawberries. Further exploration of these regulatory mechanisms holds promise for developing strawberry varieties enriched in anthocyanin, which not only enhances the fruit’s growth characteristics but also augments its nutritional value, benefiting human health.

Currently, through principal component analysis and variety cluster analysis, strawberry cultivars can be categorized into three rich anthocyanin types (RA), six high anthocyanin types (HA), six moderate anthocyanin types (MA), and two low anthocyanin types (LA) based on total anthocyanin content and anthocyanin species richness [13]. The diversity of fruit coloration in strawberries is a critical trait determining their market appeal and nutritional quality. The selective accumulation of specific anthocyanin subtypes in strawberries to precisely modulate fruit coloration remains a critical challenge. Dihydroflavonol serves as the precursor for anthocyanin, and its hydroxylation patterns affect the formation of anthocyanin subtypes [15]. By selectively regulating the expression levels of flavonoid hydroxylase genes *F3′H* and *F3′5′H*, the flavonoid metabolic flux can be redirected into multiple anthocyanin subtype biosynthesis. Additionally, distinct anthocyanin subtypes exhibit unique modification. Through metabolic engineering, site-selective modifications of anthocyanin precursors can be achieved by introducing enzymes isolated from other species, such as methyltransferases and glycosyltransferases with known catalytic specificity for targeted sites, or by overexpressing the enzyme gene already identified in strawberry. This may enable the accumulation of specific anthocyanin subtypes. The emergence of CRISPR/Cas9 technology represents a significant breakthrough in genetic engineering and breeding, offering a powerful and efficient technology to edit genes. In octoploid species like cultivated strawberries, where multiple homologous alleles may exist for a single gene, CRISPR/Cas9 can modify several alleles concurrently using a single guide RNA (sgRNA) or a limited number of sgRNAs [127]. Moreover, CRISPR/Cas9-induced genetic alterations can be reliably passed on to subsequent generations, ensuring stable inheritance of desired traits. Additionally, transgenes introduced via CRISPR/Cas9 can be segregated into offspring, facilitating the isolation of desired genetic modifications in breeding programs. This technology holds immense promise for accelerating the breeding of strawberries with improved traits, including enhanced anthocyanin content, by precisely targeting and modifying key genes involved in anthocyanin biosynthesis. Therefore, CRISPR/Cas9 technology has great application prospect in cultivating strawberry varieties with high anthocyanin.

When engineering anthocyanin biosynthesis, it is critical to consider potential metabolic trade-offs. For instance, increased anthocyanin accumulation can lead to the accumulation of acid, resulting in undesirable astringency [128]. Therefore, strawberry breeding necessitates the integration and balance of multiple quality traits. Hybridization of high-anthocyanin strawberry varieties with existing cultivars exhibiting superior sweetness and flavor to integrate these desirable traits is a key focus for future breeding strategies. Moreover, research suggest that plants may have evolved mechanisms that produce and control chemically unrelated pigments in distinct pathways. It is important to investigate whether analogous regulatory mechanisms exist in strawberry and elucidate the molecular mechanisms.

The study of anthocyanin accumulation in strawberries has made significant progress, but several key questions remain unanswered:

1. Incomplete regulatory network: A comprehensive understanding of the anthocyanin metabolic regulatory network, encompassing transcriptional regulatory factors, epigenetic modifications, and plant hormones, is lacking. The synergistic interactions among these factors and their precise regulatory mechanisms remain unclear.
2. Transport pathways: The specific processes involved are not fully elucidated, and the influence of transport pathways on anthocyanin accumulation in strawberries is not well studied. Further research is needed to unravel the transport mechanisms and their contributions to anthocyanin accumulation.
3. Varietal differences: There are observed differences in the regulation of anthocyanin metabolism among different strawberry varieties, but the underlying rules governing these variations have not been identified. Understanding these differences could provide valuable insights into varietal-specific anthocyanin regulation.

In summary, the study of anthocyanin accumulation in strawberries poses numerous challenges and unknowns, indicating the importance for continuing research efforts to unravel the complexities of this fascinating field.

## Data Availability

All data supporting the findings of this review are available within the article.
